# Identification and analysis of circulating long non-coding RNAs with high significance in diabetic cardiomyopathy

**DOI:** 10.1038/s41598-021-82345-7

**Published:** 2021-01-28

**Authors:** Tarun Pant, Anuradha Dhanasekaran, Ming Zhao, Edward B. Thorp, Joseph M. Forbess, Zeljko J. Bosnjak, Ivor J. Benjamin, Zhi-Dong Ge

**Affiliations:** 1 Department of Medicine, Medical College of Wisconsin, 8701 Watertown Plank Road, Milwaukee, WI 53226 USA; 2 Cardiovascular-Thoracic Surgery, Departments of Pediatrics and Surgery, Feinberg School of Medicine, Ann & Robert H. Lurie Children’s Hospital of Chicago, Northwestern University, 225 E. Chicago Avenue, Chicago, IL 60611 USA; 3 Centre for Biotechnology, Anna University, Chennai, Tamil Nadu 600025 India; 4 Division of Cardiology, Department of Medicine, Feinberg School of Medicine, Northwestern University, 300 E. Superior Avenue, Chicago, IL 60611 USA; 5 Department of Pathology, Feinberg School of Medicine, Northwestern University, 300 E. Superior Avenue, Chicago, IL 60611 USA

**Keywords:** Epigenetics, Gene ontology

## Abstract

Diabetic cardiomyopathy (DCM) lacks diagnostic biomarkers. Circulating long non-coding RNAs (lncRNAs) can serve as valuable diagnostic biomarkers in cardiovascular disease. To seek potential lncRNAs as a diagnostic biomarker for DCM, we investigated the genome-wide expression profiling of circulating lncRNAs and mRNAs in type 2 diabetic db/db mice with and without DCM and performed bioinformatic analyses of the deregulated lncRNA-mRNA co-expression network. Db/db mice had obesity and hyperglycemia with normal cardiac function at 6 weeks of age (diabetes without DCM) but with an impaired cardiac function at 20 weeks of age (DCM) on an isolated Langendorff apparatus. Compared with the age-matched controls, 152 circulating lncRNAs, 127 mRNAs and 3355 lncRNAs, 2580 mRNAs were deregulated in db/db mice without and with DCM, respectively. The lncRNA-mRNA co-expression network analysis showed that five deregulated lncRNAs, XLOC015617, AK035192, Gm10435, TCR-α chain, and MouselincRNA0135, have the maximum connections with differentially expressed mRNAs. Bioinformatic analysis revealed that these five lncRNAs were highly associated with the development and motion of myofilaments, regulation of inflammatory and immune responses, and apoptosis. This finding was validated by the ultrastructural examination of myocardial samples from the db/db mice with DCM using electron microscopy and changes in the expression of myocardial tumor necrosis factor-α and phosphorylated p38 mitogen-activated protein kinase in db/db mice with DCM. These results indicate that XLOC015617, AK035192, Gm10435, TCR-α chain, and MouselincRNA0135 are crucial circulating lncRNAs in the pathogenesis of DCM. These five circulating lncRNAs may have high potential as a diagnostic biomarker for DCM.

## Introduction

Type 2 diabetes mellitus (T2DM) is a global public health problem with the rising number of patients^1^. Individuals with T2DM have a twofold increased risk factor for cardiovascular disease, and cardiovascular disease persists as the leading cause of death in patients with T2DM worldwide^2,3^. Among all cardiovascular diseases, ischemic heart disease, stroke, and diabetic cardiomyopathy (DCM) are recognized as the utmost causes of heart failure and death in patients with T2DM^2,4,5^. Unlike well-known conditions of hypertension and ischemic heart disease, there are no specific approaches for diagnosis of DCM due to lack of clinical symptoms and signs in the early and middle stages of DCM, and moreover, there are no diagnostic biomarkers for DCM^6^.

Long non-coding RNAs (lncRNAs) are non-protein coding transcripts that exceed 200 nucleotides in length. In contrast to protein-coding RNAs, lncRNAs have limited protein-coding potential due to lack of protein domains or significant open reading frames^7^. However, lncRNAs display a striking ability to regulate gene expression^8,9^. Recently, lncRNAs have been demonstrated to be regulated in a cell- and tissue-specific manner in the cardiovascular disease^10–12^. Moreover, lncRNAs existing in human circulation and bodily fluids can serve as a valuable diagnostic biomarker in cardiovascular disease^13,14^.

Our group recently reported the expression profile and signature of cardiac lncRNAs and mRNAs associated with DCM in both human induced pluripotent stem cell-derived cardiomyocytes and T2DM mice^15,16^. The genome-wide expression profiling and characterization of circulating lncRNAs and mRNAs in DCM remain unknown. In addition, it is unclear whether regulation of circulating lncRNAs in DCM is the same as the changes in cardiac ones. The present study investigated the genome-wide profile of lncRNAs and mRNAs in the plasma of T2DM mice with and without DCM. To seek the potential lncRNAs with high potential as a diagnostic biomarker for DCM, we conducted the bioinformatic analysis of the deregulated lncRNA-mRNA co-expression network. Furthermore, we validated the findings from the bioinformatic analyses via the ultrastructural examination of myocardial samples from the T2DM mice with DCM using electron microscopy and the measurement of the expression of related myocardial proteins.

## Results

### Pathophysiological characteristics and cardiac phenotype of db/db mice

Table 1 lists the general and echocardiographic parameters of C57BL/6J and db/db mice at both 6 and 20 weeks of age. Compared with age-matched C57BL/6J controls, db/db mice at two ages displayed increased body weight, fasting blood glucose levels, and anterior and posterior wall thickness of the LV (P < 0.05, n = 12–13 mice/group). There were no significant differences in the LV chamber volumes at both end diastole and end systole between db/db and age-matched C57BL/6J mice. The LV ejection fraction (an index of LV systolic function) and mitral E/A ratio (an index of LV diastolic function) were comparable between 6-week-old db/db and C57BL/6J mice. Interestingly, although there were not significant differences in ejection fraction between 20-week-old db/db mice and C57BL/6J controls, mitral E/A ratio was significantly lower in 20-week-old db/db mice than C57BL/6J controls (P < 0.05). These results suggest that obese db/db mice develop DCM at 20 weeks of age, although they have hyperglycemia at 6 weeks of age.

**Table 1:** Pathophysiological and echocardiographic parameters of C57BL/6J and db/db mice.

	20 weeks old		6 weeks old	
	C57BL/6J	db/db	C57BL/6J	db/db
Body weight, g	20.9 ± 0.4	36.7 ± 0.9*	28.9 ± 0.7^*†^	59.2 ± 1.5^*†#^
Blood glucose, mg/dl	167 ± 24	368 ± 35*	178 ± 14^†^	322 ± 19*#
Anterior wall at end diastole, mm	0.81 ± 0.02	0.98 ± 0.03*	0.90 ± 0.04*	1.19 ± 0.05^*†#^
Anterior wall at end systole, mm	1.31 ± 0.05	1.57 ± 0.06*	1.27 ± 0.05^†^	1.66 ± 0.06^*#^
Posterior wall at end diastole, mm	0.80 ± 0.03	1.00 ± 0.06*	0.88 ± 0.03	1.23±0.05^*†#^
Posterior wall at end systole, mm	1.18±0.05	1.37 ± 0.06*	1.20 ± 0.05	1.57 ± 0.06^*#^
LV chamber volume at end diastole, µl	58 ± 4	59 ± 5	76 ± 4^*†^	65 ± 6
LV chamber volume at end systole, µl	19 ± 3	15 ± 2	28 ± 3^*†^	27 ± 2^†^
Ejection fraction, %	68 ± 3	73 ± 2	64 ± 3^†^	56 ± 4^*†^
Peak E/A ratio	1.59 ± 0.09	1.55 ± 0.05	1.60 ± 0.07	1.00 ± 0.03^*†#^

*LV* left ventricular.

*P < 0.05 versus 6-week-old C57BL/6J mice.

^†^P < 0.05 versus 6-week- old db/db mice.

^#^P < 0.05 versus 20-week-old C57BL/6J mice (n = 12–13 mice/group).

### Increased cardiomyocyte size and myocardial fibrosis in db/db mice at 20 weeks of age

**Figure 1:**
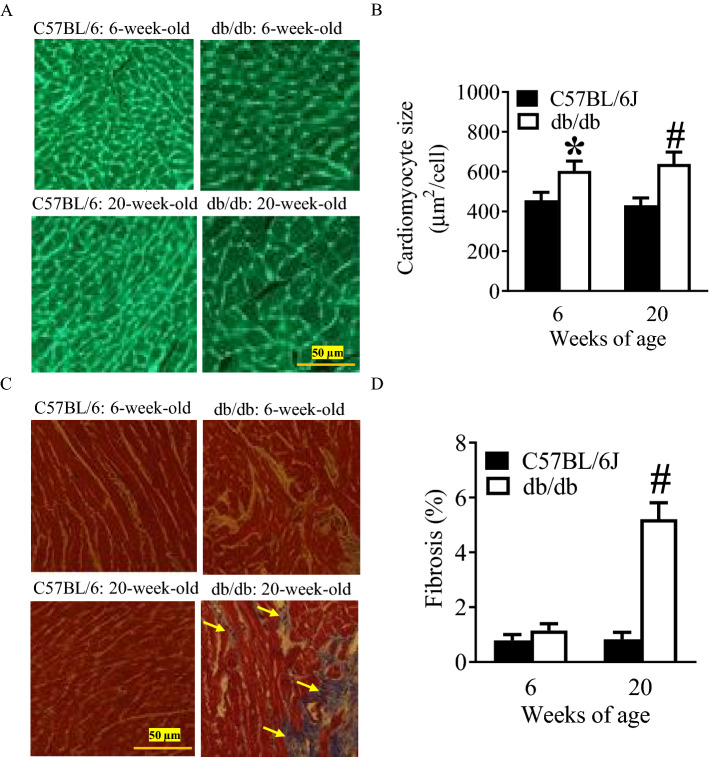
Histopathological analysis of the hearts of C57BL/6J and db/db mice at 6 and 20 weeks of age. **(A)** Representative photomicrographs of mouse hearts following a wheat germ agglutinin staining; **(B)** quantification of cardiomyocyte size; **(C)** representative photomicrographs of mouse hearts following a Masson’s trichrome staining; **(D)** quantification of myocardial fibrosis. Wheat germ agglutinin (**A**,**B**) and Masson’s trichrome (**C,D**) were used to stain left ventricular sections of C57BL/6J and db/db mice. In Masson’s trichrome staining, interstitial fibrosis was stained in blue, as indicated by yellow arrows, and ventricular muscle was stained in red. The scale bar represents 50 µm. *P < 0.05 versus C57BL/6J group at 6 weeks old; ^#^P < 0.05 versus C57BL/6J group at 20 weeks old (n = 10/group).

Figure 1 shows the histopathological changes of db/db mice at 6 and 20 weeks of age. Wheat germ agglutinin-stained hearts display an increase in cardiomyocyte size in db/db mice at both 6 and 20 weeks of age (P < 0.05, n = 10/group) compared with C57BL/6J control mice (Fig. 1A,B). Masson’s trichrome was used to stain mouse hearts to identify fibrosis (Fig. 1C). Myocardial fibrosis was not significantly increased in db/db mice at 6 weeks of age compared with C57BL/6J mice (P > 0.05, n = 10/group) (Fig. 1D). Interestingly, it was significantly more in 20-week-old db/db mice than C57BL/6J mice and 6-week-old db/db mice (Fig. 1D). These data indicate that db/db mice at 20 weeks of age have cardiac hypertrophy and myocardial fibrosis.

### Differential expression profile of circulating lncRNAs in db/db mice with and without DCM

**Figure 2:**
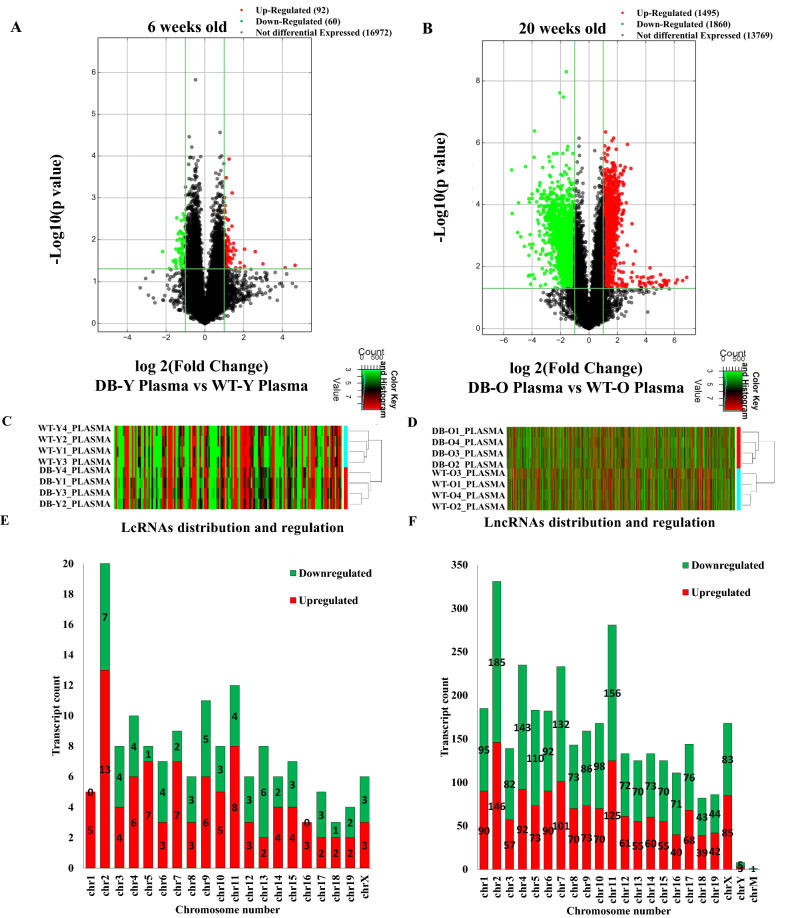
The profiling of deregulated lncRNAs in the plasma of db/db mice with and without diabetic cardiomyopathy compared with controls. **(A,B)** Volcano plots showing differently expressed lncRNAs in 6 and 20-week-old db/db mice, respectively, compared with controls. The db/db mice develoed early diabetic cardiomyopathy at 20 weeks old. The red and green points represent up- and down-regulated lncRNAs, respectively. The horizontal green line depicts *P ≤ 0.05 (n = 12–13 mice/group), whereas the vertical green line shows a twofold change of up and down. **(C,D)** Hierarchical cluster analysis presenting differentially expressed lncRNAs between 6 and 20-week-old db/db and control mice, respectively. Colors of red and green represent up- and down-regulated lncRNAs with changes larger than twofold, respectively. **(E,F)** Chromosomal distribution of deregulated lncRNAs in 6 and 20-week-old db/db mice, respectively. Colors of green and orange represent up- and down-regulated lncRNAs.

Figure 2 shows the differential expression profiles of circulating lncRNAs in 6- and 20-week old db/db mice. Compared with the age-matched controls, 152 and 3,355 lncRNAs were deregulated in db/db mice at 6 and 20 weeks of age, respectively (Fig. 2A,B). Among them, 33 lncRNAs overlapped. Compared with 6-week-old db/db mice, 19/33 lncRNAs were upregulated, and 14/33 down-regulated in 20-week-old db/db mice (Figure S1). Figure 2C,D are hierarchical clustering depicting the heterogeneity of deregulated lncRNAs between db/db and C57BL/6J mice at 6 and 20 weeks of age. To understand the lncRNA functions, we analyzed the chromosomal location and classification of deregulated lncRNAs. Majority of the deregulated lncRNAs in both 6- and 20-week-old db/db mice were mapped on chromosome 2 (Fig. 2E,F). In 6-week-old db/db mice, 13 lncRNAs on chromosome 2 were upregulated, accounting for 14.1% of all 92 upregulated lncRNAs, and 7 lncRNAs on chromosome 2 were downregulated, constituting 11.7% of all 60 downregulated lncRNAs. In the 20-week- old db/db mice, 146 lncRNAs on chromosome 2 were upregulated, constituting 9.8% of all 1,495 upregulated lncRNAs, and 185 lncRNAs on chromosome 2 were downregulated, accounting for 9.9% of all 1,860 down-regulated lncRNAs.

The lncRNA can be categorized into intergenic, intronic antisense, natural antisense, bidirectional, exon sense overlapping, and intron sense overlapping groups based on their relative position to the coding gene^10^. In db/db mice at 6 weeks of age, 40% of deregulated lncRNAs were intergenic, 32% were exon sense overlapping, and 11% were intronic antisense (Figure S2A). However, in db/db mice at 20 weeks of age, intergenic, exon sense overlapping, and intronic antisense lncRNAs accounted for 46%, 32%, and 7%, respectively (Figure S2B).

### Differential expression profile of circulating mRNAs in db/db mice with and without DCM

**Figure 3:**
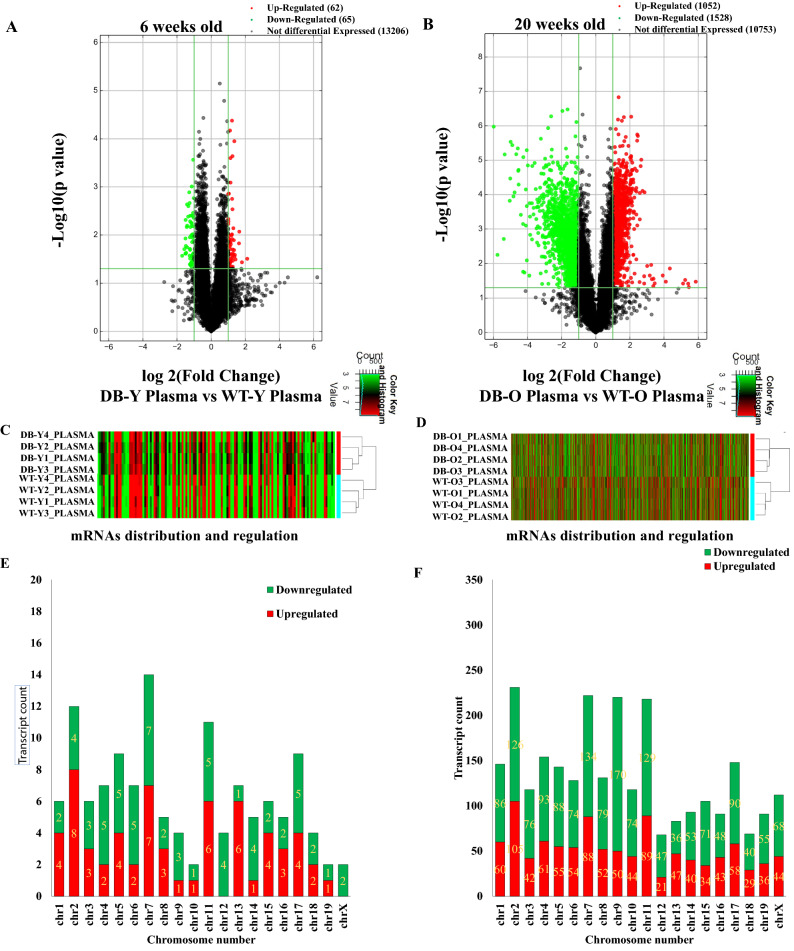
Differential expression Profile of circulating mRNA in db/db mice with and without early diabetic cardiomyopathy compared with controls. **(A,B)** Volcano plots showing deregulated mRNAs in db/db mice at 6 and 20 weeks of age, respectively compared with age-matched controls. The red and green points represented up- and down-regulated mRNAs, respectively. The horizontal green line depicts *P ≤ 0.05, whereas the vertical green line shows a twofold change of up and down. **(C,D)** Hierarchical clustering analysis demonstrating differently expressed mRNAs between 6 and 20-week-old db/db mice and controls, respectively. Colors of red and green represented up- and down-regulated mRNAs with changes larger than twofold, respectively. **(E,F)** Chromosomal distribution of deregulated circulating mRNAs in 6 and 20-week-old db/db mice, respectively. Colors of green and orange represented up- and down-regulated mRNAs.

We examined 24,881 mRNA transcripts in the plasma of db/db mice. Compared with age- and gender-matched controls, 62 and 1052 mRNAs were upregulated in the plasma of 6- and 20-week-old db/db mice, respectively; whereas 65 and 1528 mRNAs were downregulated in the plasma of 6- and 20-week-old db/db mice, respectively (fold change ≥ 2.0, P ≤ 0.05) (Fig. 3A,B). Figure 3C,D are the hierarchical clustering of mRNAs showing the heterogeneity of deregulated mRNAs between 6- and 20-week-old db/db mice and age-matched controls. Deregulated mRNAs distributed in 20 chromosomes in both 6- and 20-weeks-old db/db mice. Among 20 chromosomes, chromosome 7 and 2 had the maximum number of aberrantly expressed mRNAs in db/db mice at 6 and 20 weeks of age, respectively (Fig. 3E,F). Compared with age-matched controls, 7 mRNAs in chromosome 7 and 105 in chromosome 2 were up-regulated in db/db mice at 6 and 20 weeks of age, respectively; whereas 7 mRNAs on chromosome 7 and 126 on chromosome 2 were down-regulated in db/db mice at both 6 and 20 weeks of age, respectively.

### Bioinformatic analysis of lncRNA-mRNA co-expression network

**Figure 4:**
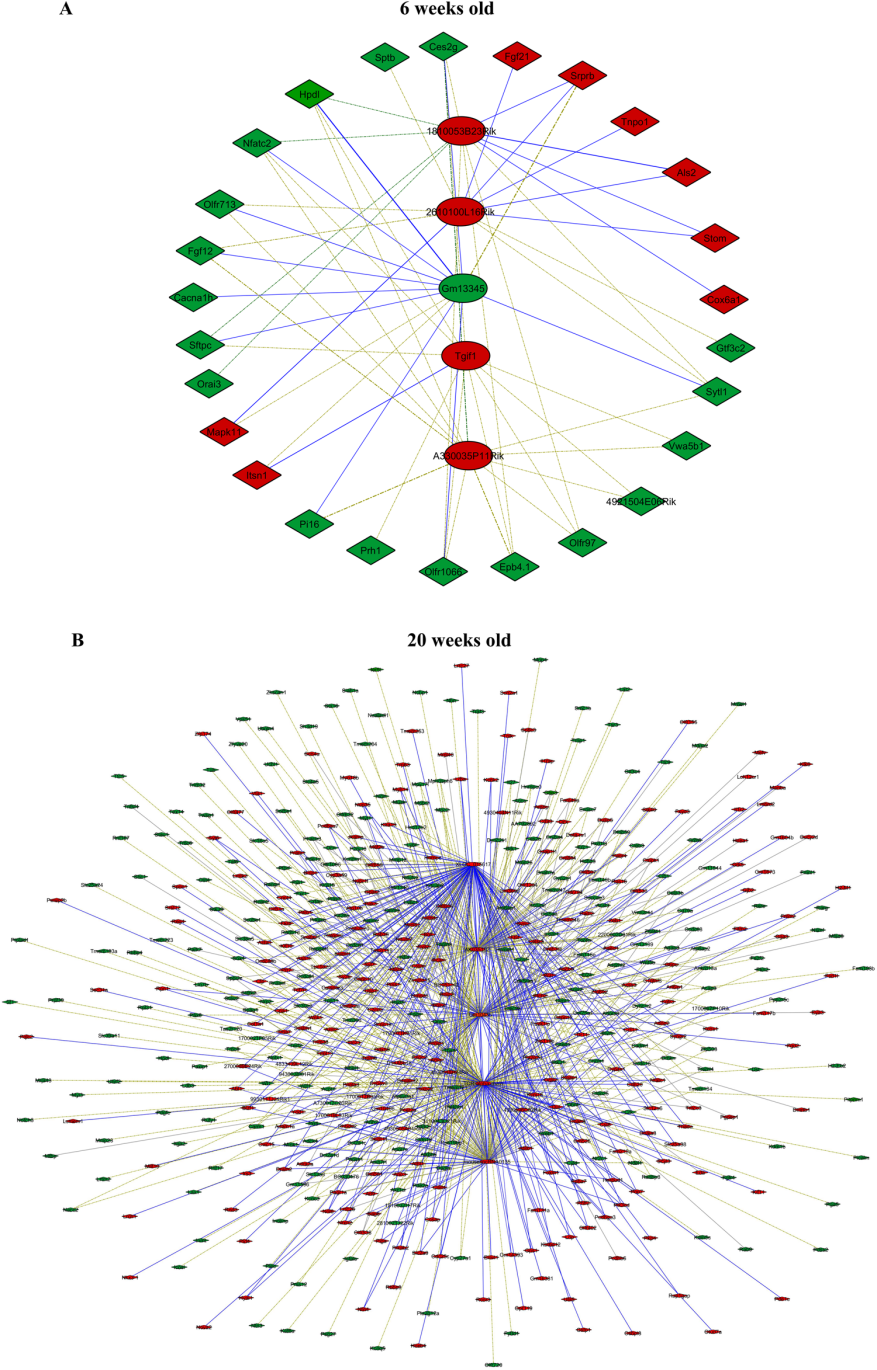
Connections of five deregulated circulating lncRNAs with mRNA transcripts in db/db mice with and without diabetic cardiomyopathy. **(A)** The network of lncRNA-mRNA co-expression in 6-week-old db/db mice; **(B)** the network of lncRNA-mRNA co-expression in 20-week-old db/db mice. The network represents the co-expression correlations between the significantly differentially expressed lncRNAs and mRNA transcripts. Five lncRNAs having maximum connections with differentially expressed genes were taken to construct the co-expression network (Pearson correlation > 0.995 or <  − 0.995 and P < 0.05). Circles, squares, and V shapes indicate lncRNA transcripts, transcription factors, and mRNA transcripts, respectively. Solid arrows and dashed lines indicate positive and negative correlation, respectively, whereas red and green colors represent up- and down-regulated transcripts. The width of the line is based on Person’s value (stronger correlation corresponds to more width) and color of the line depicts the significance. Nodes indicate lncRNAs or mRNAs.

**Figure 5:**
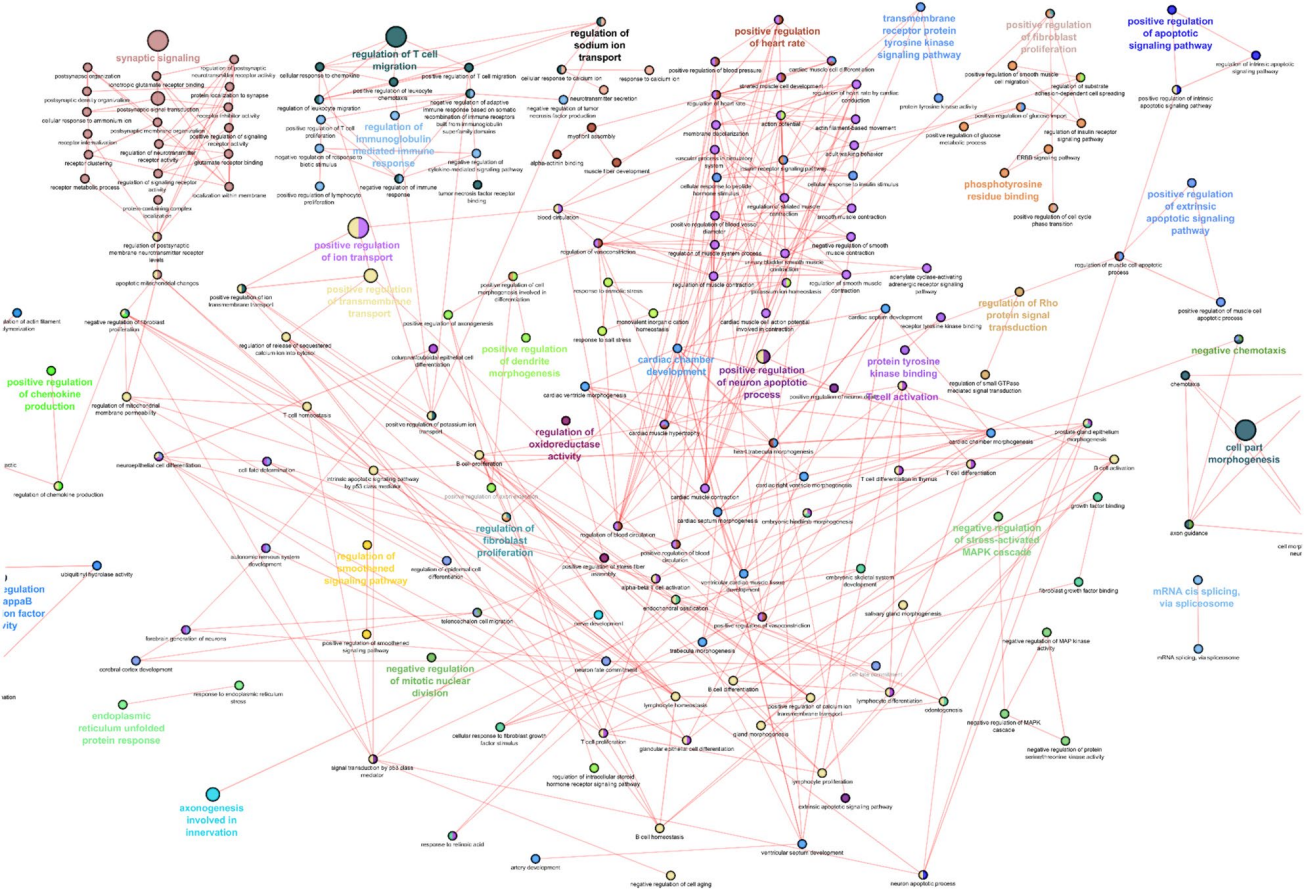
Gene ontology (GO) analysis of top 5 deregulated circulating lncRNAs in db/db mice with diabetic cardiomyopathy. The network represents the GO pathway terms specific for mRNA genes having co-expression relationship with the significantly differentially expressed top 5 lncRNAs having maximum connections in the co-expression network (Pearson correlation > 0.995 or <  − 0.995 and P < 0.05) at 20 weeks of ag. Functionally grouped networks with GO terms as node are grouped based on kappa score level (> 0.03), the most significant groups are shown. The node size represents the term enrichment significance. Different GO clusters are shown in different color whereas functionally related groups partially overlap. The color gradient represents the gene proportion of each cluster associated with that term. Nodes indicate lncRNAs or mRNAs.

Significantly co-expressed lncRNAs-mRNAs (Pearson correlation > 0.995 or <  − 0.995 and P < 0.05) were identified and assembled into co-expression networks. Figure 4 shows the co-expression network of five lncRNAs having the maximum number of connections with mRNAs. In 6-week-old db/db mice, the top 5 deregulated circulating lncRNAs, 1810053B23Rik, 2610100L16Rik, Gm13345, Tgif1, and A330035P11Rik, related to 26 mRNAs (Fig. 4A). These 26 RNAs were not associated with the gene ontology (GO) terms that are important in biological process, cellular components, and molecular function. In 20-week-old db/db mice, the top 5 deregulated circulating lncRNAs, XLOC015617, AK035192, Gm10435, TCR-α chain, and MouselincRNA0135, were linked with 522 mRNAs (Fig. 4B). These lncRNAs were correlated with many important GO terms in biological process, cellular components, and molecular function, including the development and motion of myofilaments, regulation of inflammatory and immune responses, and apoptosis (Pearson correlation > 0.995 or <  − 0.995 and P < 0.005) (Fig. 5).

### Expression of myocardial lncRNAS in db/db mice with DCM

Real-time quantitative reverse transcriptional-polymerase chain reaction (qRT-PCR) was used to measure the expression levels of five core lncRNAs in the hearts of 20-week-old C57BL/6 and db/db mice. The qRT-PCR analysis revealed that the expression of XLOC015617, AK035192, Gm10435, TCR-α chain, and MouselincRNA0135 was significantly up-regulated in db/db mouse hearts compared with controls (Figure S5). These results suggest that myocardial lncRNAs are dysregulated in DCM.

### Altered myocardial myofilaments and mitochondria in db/db mice with DCM

**Figure 6:**
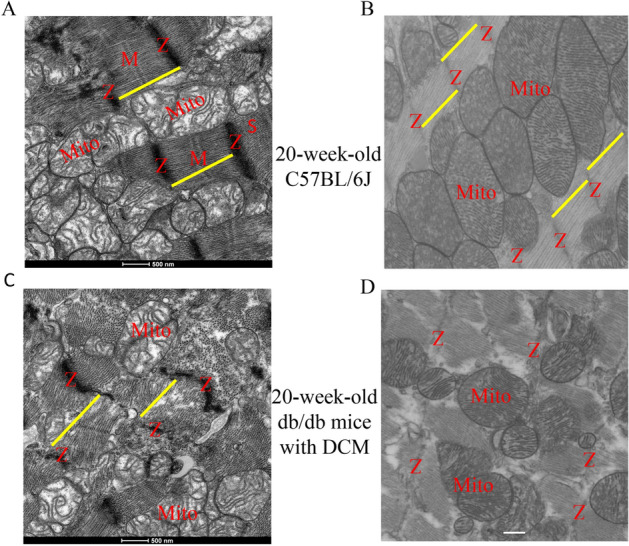
Transmission electron microscopy micrographs of myocardial myofilaments and mitochondria in db/db mice with diabetic cardiomyopathy and controls. **(A)** Non-diabetic control ventricle showed well-defined sarcomeres (yellow line) with distinguishable Z-lines (Z) and M-lines (M) throughout the tissue; **(B)** non-diabetic control ventricle showed well-organized sarcomeres and mitochondria (Mito); **(C)** the sarcomeric structure of db/db mice with DCM was less organized, with blurred Z-lines and absent M lines; **(D)** in db/db mice with DCM, Z-lines were disarrayed, the sarcomeres were damaged, and mitochondrial volumes were decreased. Scar bar: 500 nm.

The above GO analyses of both deregulated mRNAs (Figure S3) and the lncRNA-mRNA co-expression network revealed that myofilaments were altered in DCM. To validate this finding, we observed the ultrastructure of left ventricular myocardium in db/db mice with DCM, focusing on myofilaments and mitochondria. The transmission electronic microscopy analysis displayed that the ventricle of non-diabetic C57BL/6J mice at 20 weeks of age showed well-defined sarcomeres and mitochondria, with distinguishable Z-lines and M-lines throughout the tissue (Fig. 6A,B). However, the left ventricular myocardium of db/db mice at 20 weeks of age had a sarcomeric defect in the myocardial tissue of the 20-week-old db/db mice compared with the age-matched controls. Ultrastructurally, the LV myocardium of db/db mice with DCM showed disarray of normal sarcomeric order or highly disorganized sarcomeres and decreases in mitochondrial volumes, with poorly defined Z-lines and M-lines (Fig. 6C,D).

### Changed myocardial TNF-α and p38 MAPK proteins in db/db mice with DCM

**Figure 7:**
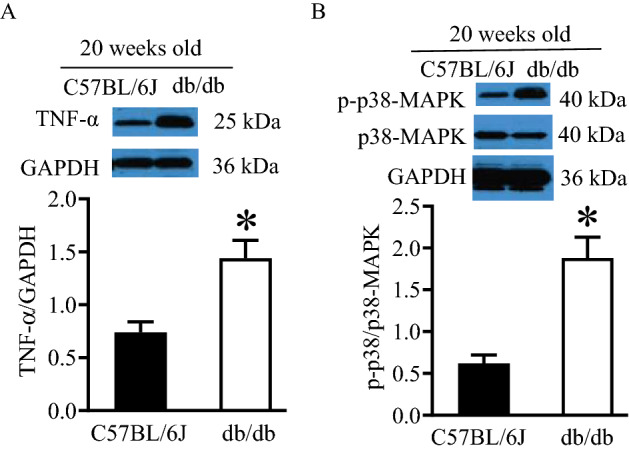
Up-regulated expression of tumor necrosis protein-α (TNF-α) and phosphorylated p-38 mitogen-activated protein kinase (p-p38 MAPK) in the myocardium of db/db mice with diabetic cardiomyopathy. **(A)** The expression of myocardial TNF-α. Top: representative Western blot bands of TNF-α and glyceraldehyde-3-phosphate dehydrogenase (GAPDH). Bottom: the ratio of TNF-α/GAPDH; **(B)** the expression of myocardial p-p38 MAPK and p38 MAPK. Top: representative western blot bands of p-p38 MAPK, p38 MAPK, and GAPDH as control. Bottom: the ratio of p-p38-MAPK/p38 MAPK. *P < 0.05 versus C57BL/6J groups (n = 5 mice/group). The Western blot bands were cropped from Figure S6, which were pointed by red arrows. In Figure S6, the original images of Western blot bands included C57BL/6J and db/db mice at 6 weeks of age in addition to 20-week-old mice, and several Western blot experiments were put on a big membrane.

The bioinformatic analysis revealed the importance of tumor necrosis factor (TNF) and mitogen-activated protein kinase (MAPK)-signaling pathways in DCM for deregulated circulating lncRNAs (Figs. 5 and S4). To validate this finding, we measured the expression of TNF-α and p38 MAPK proteins in the heart of db/db mice with DCM. As shown in Figs. 7 and S6, the ratio of TNF-α/GAPDH and phosphorylated p-38 MAPK (p-p38 MAPK)/p38 MAPK were significantly increased in db/db mice with DCM compared with non-diabetic controls (P < 0.05, n = 5 mice/group).

## Discussion

The results of the present study demonstrate that db/db mice develop diabetes and DCM at 6 and 20 weeks of age, respectively. We identified 3355 circulating lncRNAs and 2580 mRNAs were aberrantly expressed in db/db mice with DCM. Among all 3,355-deregulated circulating lncRNAs in DCM, XLOC015617, AK035192, Gm10435, TCR-α chain, and MouselincRNA0135 have the maximum connections with differently expressed mRNAs. Bioinformatic analysis reveals that they are highly associated with the development and motion of myofilaments, inflammation, immunity, and apoptosis. These 5 circulating lncRNAs may be the core lncRNAs with high significance in DCM.

T2DM primarily occurs because of obesity and lack of exercise. It is characterized by hyperglycemia, insulin resistance, and relative lack of insulin, frequently accompanied by left ventricular hypertrophy in the patients^17,18^. In the present study, the db/db mice at 20 weeks of age had decreased mitral E/A ratio and increased cardiomyocyte size and myocardial fibrosis. These results indicate that the db/db mice at 20 weeks of age develop DCM.

In the present study, 3355 differently expressed circulating lncRNAs in db/db mice with DCM were distributed on 20 chromosomes. Compared with other chromosomes, chromosome 2 had the maximum number of deregulated lncRNAs in db/db mice with and without DCM. These findings are consistent with our previous study showing the predominant distribution of cardiac lncRNAs on chromosome 2 in db/db mice with and without DCM^16^. Previous studies have shown the hereditary influence on diabetic prevalence^19,20^. Individuals with a history of parental diabetes were more susceptible to develop the disease than those having no background^21,22^. Interestingly, previous studies have indicated that chromosome 2 holds the genes that are susceptible to diabetes^23–25^. Taken together, chromosome 2 may be more likely to carry lncRNAs susceptible to the pathology of DCM.

LncRNAs tend to show more diversified mechanisms of action as most lncRNAs are involved in regulating gene expression via processes like chromatin modification or DNA methylation, targeting transcription factors or blocking transcription depicting both their *cis* and *trans* regulating potential^26^. We constructed the lncRNA-mRNA co-expression network to identify key lncRNAs associated with DCM. Our co-expression network showed that the lncRNA, XLOC015617, AK035192, Gm10435, TCR-α chain, and MouselincRNA0135, had the highest number of connection with the mRNA transcripts. These 5 circulating lncRNAs were significantly elevated in the db/db mouse hearts with DCM. Bioinformatic analyses revealed that they were widely associated with altered myofilaments, inflammation, immunity, and apoptosis which play a vital role in the pathogenesis of DCM^27–29^. Despite unclear functions and signaling transduction pathways, the dysregulation of these five circulating lncRNAs may be of crucial importance since they have the maximum association with other mRNAs.

The lncRNA-mRNA co-expression network revealed that cardiac myofilaments were altered in DCM. A previous study reported that myocardial myofilaments were damaged in DCM^30^. Thus, we examined the ultrastructure of the myocardium of 20-week-old db/db mice. The transmission electronic microscopy analysis indicated that the sarcomeres in the ventricular myocardium were highly disorganized, with poorly defined Z-line and M-lines in db/db mice with DCM. Myofilaments consist of thin filament proteins, which include actin, tropomyosin, and the troponin complex, and thick filament proteins including myosin, as well as a number of accessory proteins such as myosin binding protein C^31^. Cardiac contraction and relaxation require the integrated activity of highly coordinated protein–protein interactions among myofilament proteins in the sarcomere^32,33^. Collectively, dysregulated lncRNAs may contribute to damaged myofilaments in DCM.

The GO analysis of the five core lncRNAs revealed that they were associated with altered myofilament, inflammation, immunity, and apoptosis which contribute to the pathogenesis of DCM^28,29,34^. TNF is produced predominantly by macrophages, and elevated production of TNF-α in DCM facilitates myocardial inflammation, fibrosis, apoptosis^35,36^. p38 MAPK is phosphorylated (activated) in DCM, leading to myocardial inflammation, fibrosis, apoptosis, and dysfunction via its signaling cascade^37–39^. Moreover, both enhanced expression of TNF-α and the activation of p38 MAPK are associated with a change in mitochondrial function in DCM^40,41^. Thus, we determined the expression of expression of myocardial TNF-α and p38 MAPK in DCM. Our Western blot results indicate that the expression of TNF-α proteins was significantly elevated, and p38 MAPK was phosphorylated in DCM. These results are consistent with previous reports demonstrating that both TNF-α- and p38 MAPK-signaling pathways play important roles in the pathogenesis of DCM^35,38^. Taken together, dysregulated circulating lncRNAs are associated with important intracellular signaling pathways involved in the pathogenesis of DCM.

The lack of specific biomarkers coupled with the fact that the earlier stages of DCM are mostly asymptomatic makes detecting DCM a challenge in clinical practice^42^. The possibility to easily detect differently expressed lncRNAs that remain stable in plasma opens up the opportunity to use them as biomarker for DCM. This notion is supported by a recent study showing that the circulating intergenic lncRNA, LIPCAR, is an independent predictor of diastolic dysfunction in asymptomatic diabetic patients^13^. In the present study, our microarray data indicate that XLOC015617, AK035192, Gm10435, TCR-α chain, and MouselincRNA0135 are crucial circulating lncRNA in DCM. Further studies are needed to investigate whether they can serve as the biomarker of diagnosis and prognosis for DCM.

In conclusion, the present study indicates that many circulating lncRNAs and mRNAs are differentially expressed in DCM. We identify XLOC015617, AK035192, Gm10435, TCR-α chain, and MouselincRNA0135 as core lncRNAs with high significance in DCM. Bioinformatic analyses reveal that they are widely associated with myofilaments, immunity, and inflammation. To find the suitable biomarker for DCM, future studies will examine whether these five circulating lncRNAs can serve as diagnostic biomarker for DCM.

## Materials and methods

### Animals

Db/db (B6.BKS(D)-Lepr^*db*^/J*, male*) and C57BL/6J control mice were purchased from The Jackson Laboratory (Bar Harbor, ME, USA). The animal care and all experimental procedures were performed in accordance with the ARRIVE guideline, and experimental protocols were approved by the Institutional Animal Care and Use Committee at the Medical College of Wisconsin (Milwaukee, WI, USA) or Northwestern University (Chicago, IL, USA). All methods were carried out in accordance with relevant guidelines and regulations.

### Measurements of blood glucose

Mice were fasted for 6 h (12 mice/group). Blood glucose was measured with a blood gas analyzer (ABL-725 Radiometer, Radiometer America Inc., Westlake, OH, USA), as described^37^.

### Thoracic echocardiography

The structure and function of the LV were evaluated with a VisualSonics Vevo 3100, as described^43,44^.

### Histopathological analysis of mouse hearts

The LV of C57BL/6J and db/db mice were stained with wheat germ agglutinin and Masson’s trichrome to quantitate cardiomyocyte size and myocardial fibrosis, respectively, as described^37,45^.

### RNA extraction

The heparinized blood was centrifuged to obtain plasma. Total RNA was isolated using the TRIZOL reagent according to the manufacturer’s instruction (Invitrogen, Carlsbad, USA)^16^. The extracted RNA was further been processed for microarray, as described^16^.

### RNA microarrays and bioinformatic analysis

LncRNA and mRNA microarrays were carried out by Arraystar Inc. (Rockville, MD) using the Mouse LncRNA Microarray V3.0, a Agilent Array Platform (Agilent Technologies, Inc., Santa Clara, CA, USA)^16^. LncRNAs and the differentially expressed protein-coding genes were identified, and fold changes as well as *P* values from the statistics t-test were calculated. The up- or down-regulated lncRNAs were set as fold change ≥ 2.0 and *P* value ≤ 0.05 and shown in volcano plots and heat maps. GO and Kyoto Encyclopedia of Genes and Genomes (KEGG) analysis were applied to explore the potential roles that the differentially expressed mRNAs played in biological pathways or GO terms, including the following three categories: biological process, cellular component, and molecular function^16^.

### Construction and analysis of lncRNA-mRNA co-expression network

The lncRNA-mRNA co-expression networks were constructed using Cytoscape software (v3.4.0), according to the normalized signal intensity of individual genes^16^. A co-expression analysis was performed by associating the expression profiles of deregulated lncRNAs with deregulated mRNAs.

### qRT-PCR analysis of myocardial lncRNAs

Total RNA from the LV was extracted using Triazol reagent according to the protocol of the manufacturer (Qiagen, Valencia, CA). qRT-PCR was used to analyze the expression of myocardial lncRNAs, as described^16,46^.

### Electron microscopy of myocardial ultrastructure

The LV of mice was cut into 1 mm fragments and fixed in 2.5% glutaraldehyde (0.2 M cacodylate buffer, pH 7.4) for 4 h at 4 °C, and post-fixed in 1% osmium tetroxide, as described^47^. The ultrathin sections stained with toluidine blue were examined under a JEM-2100 transmission electron microscope (JEOL, Peabody, MA, USA) to identify myofilaments and mitochondria.

### Western blot analysis of protein expression

The LV homogenates that contained 50 µg of protein were applied to 7.5% sodium dodecyl sulfate (SDS)-polyacrylamide gel and subjected to immunoblot analysis by incubation with primary antibodies against against the pro-inflammatory cytokine TNF-α, p38 MAPK, phosphorylated Thr180/Thr182 p38 MAPK (p-p38 MAPK), glyceraldehyde-3-phosphate dehydrogenase (GAPDH) at 4 °C overnight, as described^46,48^. GAPDH was used as a reference protein for TNF-α and p38 MAPK. The membrane was then incubated with the appropriate anti-mouse or anti-rabbit secondary antibody.

### Statistics

One-way ANOVA with repeated measures was used to evaluate differences in the LV hemodynamic parameters from Langendorff-perfused hearts. Non-parametric Mann Whitney test was used to compare the gene expression between two groups. Benjamini–Hochberg FDR (cut off 0.05) was applied for multiple-testing correction. A value of *P* less than 0.05 (two tailed) was considered statistically significant.

## Supplementary Information

Supplementary Information.

## Data Availability

The data that support the findings in this study are available from the corresponding author on request.
