# Supplementary material for: Identification and analysis of circulating long non-coding RNAs with high significance in diabetic cardiomyopathy
Source: Sci Rep. 2021 Jan 28;11:2571. doi: 10.1038/s41598-021-82345-7 (PMC7843621; doi:10.1038/s41598-021-82345-7)
Supplement: Supplementary file 1 — Supplementary Information. [file 41598_2021_82345_MOESM1_ESM.docx]

Supplementary Materials for

**Identification and analysis of circulating long non-coding RNAs**

**with high significance in diabetic cardiomyopathy**

Tarun Pant, Anuradha Dhanasekaran, Ming Zhao, Edward B. Thorp,

Joseph M. Forbess, Zeljko J. Bosnjak, Ivor J. Benjamin, and Zhi-Dong Ge*

*Corresponding author. E–mail: [zhi-dong.ge@northwestern.edu](mailto:zhi-dong.ge@northwestern.edu) (Z.-D.G.)

The Supplementary Materials includes:

Supplementary materials and methods

Figure S1. Overlapping deregulated lncRNAs in db/db mice at 6 and 20 weeks of age.

Figure S2. Classification of deregulated circulating lncRNAs in db/db mouse hearts with and without diabetic cardiomyopathy.

Figure S3. Gene ontology (GO) enrichment of deregulated circulating mRNAs in db/db mice with and without diabetic cardiomyopathy.

Figure S4. Kyoto Encyclopedia of Genes and Genomes (KEGG) pathway analysis of deregulated circulating mRNAs in db/db mice with and without diabetic cardiomyopathy.

Figure S5. Expression of myocardial lncRNAs in C57BL/6J and db/db mice at 20 weeks of age.

Figure S6. Representative Western blot bands showing the expression of myocardial TNF-α, p-p38-MAPK, p38 MAPK, and GAPDH as control in C57BL/6J and db/db mice at both 6 and 20 weeks of age.

**Materials and Methods**

## ****Animals****

**Obese male T2DM B6.BKS(D)-Lepr*^db^*/J (*db/db*) and C57BL/6J control mice were purchased from The Jackson Laboratory (Bar Harbor, ME, USA). The animals were kept on a 12-h light-dark cycle in a temperature-controlled room.** T**he animal care and all experimental procedures were performed in accordance with the ARRIVE guideline**, and **experimental protocols were approved by the Institutional Animal Care and Use Committee at the Medical College of Wisconsin (Milwaukee, WI, USA) or Northwestern University (Chicago, IL, USA). All methods were carried out in accordance with relevant guidelines and regulations.**

We previously reported that db/db mice at 6 weeks of age had T2DM with preserved cardiac function but developed DCM at 20 weeks of age^1^. In the present study, the db/db mice were assigned to 2 experimental groups: T2DM without DCM (db/db mice at 6 weeks of age) and T2DM with DCM (db/db mice at 20 weeks of age). Age- and gender-matched C57BL/6J mice were used as controls.

## Measurements of blood glucose

C57BL/6J and db/db mice at either 6 or 20 weeks of age were fasted for 6 h (12 mice/group). Under the anesthesia of 80 mg/kg pentobarbital, a **thoracotomy was performed, and the left ventricle (LV) was punctured with a 27-gauge needle with a syringe, as described previously^2^. Blood glucose was measured with a blood gas analyzer (ABL-725 Radiometer, Radiometer America Inc., Westlake, OH, USA).**

**Histopathological analysis of mouse hearts**

Mouse hearts were excised from isoflurane-euthanized mice, washed in PBS, fixed overnight in 4% paraformaldehyde, and embedded in paraffin, as described^2^. All sections were cut from the midpoint of the left ventricle and deparaffinized and hydrated using graded concentrations of ethanol to deionized water. For the measurement of cardiomyocyte size, tissue sections were stained with a fluorescence Oregon Green 488–labeled wheat germ agglutinin.^3^ Images were obtained using an Olympus fluorescence microscope at 200× magnification, and cardiomyocyte surface area measured using ImageJ software (Figure 2A). For the measurement of myocardial fibrosis, 5-μm sections were stained with Masson’s trichrome. Fibrosis was stained in blue, whereas cardiac myocytes were stained in red, as shown in Figure 2C.

**Thoracic echocardiography**

Mice were sedated by the inhalation of 1.5 % isoflurane and oxygen (n=12-13 mice/group). Non-invasive echocardiography was performed with a VisualSonics Vevo 3100 Imaging System (Toronto, Canada), as we previously described^4,5^. Left ventricular dimensions and ejection fraction were measured by two-dimension guided M-mode method. Pulsed Doppler waveforms recorded in the apical-4-chamber view were used for the measurements of the peak velocities of mitral E (early mitral inflow) and A (late mitral inflow) waves.

## RNA extraction

Pentobarbital-anesthetized C57BL/6J and db/db mice underwent thoracotomy, **and the LV was punctured with a 27-gauge needle** with a syringe pre-treated with heparin to prevent coagulation of blood in the syringe (n=12-13 mice/group). The heparinized blood was centrifuged to obtain plasma. Total RNA was isolated using the TRIZOL reagent according to the manufacturer’s instruction (Invitrogen, Carlsbad, USA)^1^. RNA quality and quantity were determined using a Nano-Drop ND-1000 spectrophotometer (NanoDrop Technologies, Wilmington, DE, USA). Total RNA was suspended in 30 µl of nuclease-free water and stored at -80°C. The extracted RNA was further been processed for microarray, as described^1^.

**RNA microarrays and bioinformatic analysis**

LncRNA and mRNA microarrays were carried out by Arraystar Inc. (Rockville, MD) using the Mouse LncRNA Microarray V3.0, an Agilent Array Platform (Agilent Technologies, Inc., Santa Clara, CA, USA)^1^. This microarray contained 35,923 lncRNAs collected from the National Center for Biotechnology Information RefSeq, UCSC, Ensembl, RNAdb2.0, Fantom3, ncRNA expression database, and from previous publications. A total of 24,881 coding transcripts were extracted based on the Collaborative Consensus Coding Sequence (CCDS) public source. The total RNA was converted to complementary DNA (cDNA) followed by hybridization to Arraystar custom lncRNA and mRNA probes. Positive probes for housekeeping genes and negative probes were also printed onto the array for hybridization quality control. The same quantity of total RNA samples was used in each group. The input cDNA quantity (1 µg) was kept the same for all samples, so that different gene expression was based on RNA quantity. A panel of genes with a consistent expression across samples was used to normalize data. LncRNAs and the differentially expressed protein-coding genes were identified, and fold changes as well as *P* values from the statistics t-test were calculated. The up- or down-regulated lncRNAs were set as fold change ≥2.0 and P value ≤0.05 and shown in volcano plots and heat maps.

Hierarchical clustering analysis of samples based on lncRNA expression was performed using the R package “heatmap” with the “ward.D2” method (software 2.15). Gene ontology (GO) and Kyoto Encyclopedia of Genes and Genomes (KEGG) analysis were applied to explore the potential roles that the differentially expressed mRNAs played in biological pathways or GO terms, including the following three categories: biological process, cellular component, and molecular function^1^.

## Construction and analysis of lncRNA-mRNA co-expression network

To reveal potential association of differentially expressed lncRNAs with mRNAs in T2DM and DCM, we constructed the lncRNA-mRNA co-expression networks using Cytoscape software (v3.4.0), according to the normalized signal intensity of individual genes^1^. Briefly, microarray data were pre-processed by using the average expression value of all transcripts expressed from the same gene (both mRNA and lncRNA). The data were then screened for differentially expressed lncRNAs and mRNAs whose expression levels positively or negatively correlated. For each pair of lncRNA-mRNA, *Pearson* correlation test was conducted to detect significant correlation. Only strongly correlated (r^2^≥-0.9, P<-0.01) pairs were used to construct the networks and generate visual representations. In these representations, each gene corresponded to a node, and the color of nodes represented the up-regulated or down-regulated expression of the specific gene in the microarray data. A co-expression analysis was performed by associating the expression profiles of deregulated lncRNAs with deregulated mRNAs. This association was based on the information of the lncRNA-associated coding regions, which was supplied by the microarray analysis.

**Real-time quantitative reverse transcriptional-polymerase chain reaction analysis of myocardial lncRNAs**

To confirm the reliability of the lncRNA and mRNA microarray data, we selected 5 core lncRNAs that exhibited significant changes in the plasma for validation using quantitative reverse transcriptional-polymerase chain reaction (qRT-PCR). Total RNA from the LV was extracted using Triazol Reagent, as described^1^. Chloroform was added, and samples were centrifuged to facilitate phase separation. The aqueous phase was extracted and combined with ethanol in miRNeasy Mini spin columns (Qiagen). Total RNA was eluted in RNase-free water, and the concentration of extracted total RNA was quantified by the Epoch spectrophotometer (Biotek, Winooski, VT, USA). Samples were considered pure if the A260/280 ratio was between 1.9 and 2.0. One µg of total RNA from each sample was used to generate cDNA using miScript Reverse transcriptase mix, nucleic mix, and HiFlex Buffer (Qiagen). To analyze the lncRNA expression, a master mix (25 μl/well) containing the template cDNA (4.5 ng/well), RNase-free water, and miScript SYBR Green (Qiagen), and the primers (lncRNAs or the housekeeping gene, Rnu-6) was prepared according to the manufacturer’s directions. The XLOC015617 primers forward 5’-CAGGCCCGACATGAGATAA-3’, reverse: TTTGTGCTCTGTAGGAGGAAAG; The AK035192 primers forward 5’-AGTGAATGGTAGTTGGGCGA-3’, reverse: TCGCCCAACTACCATTCACTT; The Gm10435 primers forward 5’-ACTTGGACACTCTTGCATCTC-3’, reverse: GCTGCCCTTTCACTCCTAAA. qRT-PCR was conducted using the BioRad iCycler Real-Time PCR Detection System. qRT-PCR for each sample was run in triplicate. Expression of lncRMAs was normalized by expression of Rnu-6. The relative gene expressions were calculated in accordance with the ΔΔCt method.

**Electron microscopy of myocardial ultrastructure**

**The hearts of the mice were perfused with a modified Karnovsky solution containing** 2.5% glutaraldehyde **and 2% formalin in 0.1 M phosphate-buffered saline at pH 7.4.** A portion of the LV was cut into 1 mm fragments and fixed in 2.5% glutaraldehyde (0.2 M cacodylate buffer, pH 7.4) for 4 h at 4^o^C, and post-fixed in 1% osmium tetroxide, as described^6^. Survey sections were cut at 1 μm and stained with toluidine blue. The ultrathin sections were examined under a JEM-2100 transmission electron microscope (JEOL, Peabody, MA, USA) to identify myofilaments and mitochondria.

**Western blot analysis of protein expression**

The LV from db/db or C57BL/6J mice at 20 weeks of age were homogenized in lysis buffer (Cellytic MT Mammalian Tissue/ Lysis/extraction Reagent; Sigma-Aldrich), as described^7,8^. Total protein was extracted from myocardium tissue, and protein concentrations were measured using a Pierce BCA Protein Assay Kit. Equal amounts of protein were subjected to sodium dodecyl sulfate-polyacrylamide gel electrophoresis and transferred onto nitrocellulose membranes. After blocked by with 5% defatted milk powder, the membrane was incubated with the antibody against the pro-inflammatory cytokine tumor necrosis factor-α (TNF-α) (1:2000, Cell Signaling Technology, Beverly, MA, USA), p38 mitogen-activated protein kinase (p38 MAPK) (1:1000, Cell Signaling Technology), phosphorylated Thr180/Thr182 p38 MAPK (p-p38 MAPK) (1:1000, Cell Signaling Technology), glyceraldehyde-3-phosphate dehydrogenase (GAPDH) (1:10000, Sigma-Aldrich, St, Louis, MO, USA) at 4°C overnight. Then it was washed by TBST for 3 times, incubated with horseradish peroxidase-labeled secondary antibody at room temperature for 1 hour, and washed with TBST again. GAPDH was used as a reference protein for TNF-α and p38 MAPK. Proteins were visualized with an enhanced chemiluminescence kit, and the density of protein bands obtained from the images was analyzed using Multigauge software (Fuji film). The relative densities were calculated and normalized to those of the corresponding internal reference GAPDH, and then normalized to the corresponding WT control mice, which was set to a value of 1.0.

## Statistics

Kruskal-Wallis test followed by Dunn’s test were used to compare body weight and blood glucose. Repeated-measures analysis of variance followed by Bonferroni multiple comparison test was used to evaluate differences in the LV hemodynamic parameters from Langendorff-perfused hearts. Non-parametric Mann Whitney test was used to compare the gene expression between two groups, whereas Benjamini-Hochberg FDR (cut off 0.05) was applied for multiple-testing correction. All statistical analyses were performed using GraphPad Prism 8 (GraphPad Software, Inc., La Jolla, CA, USA). A value of P less than 0.05 (two tailed) was considered statistically significant.

**Results**

**Overlapping deregulated circulating lncRNAs in db/db mice with and without DCM**

In 20-week-old db/db mice, 33 out of 3,355 deregulated lncRNAs overlapped with deregulated lncRNAs in 6-week-old db/db mice. Figure S1 lists the name and regulation of 33 overlapping deregulated lncRNAs. Among them, 19 lncRNAs were upregulated, and 14 down-regulated in 20-week-old db/db mice compared with 6-week-old db/db mice. These data suggest that the expression profiles of circulating lncRNAs from T2DM are dysregulated in DCM.

#
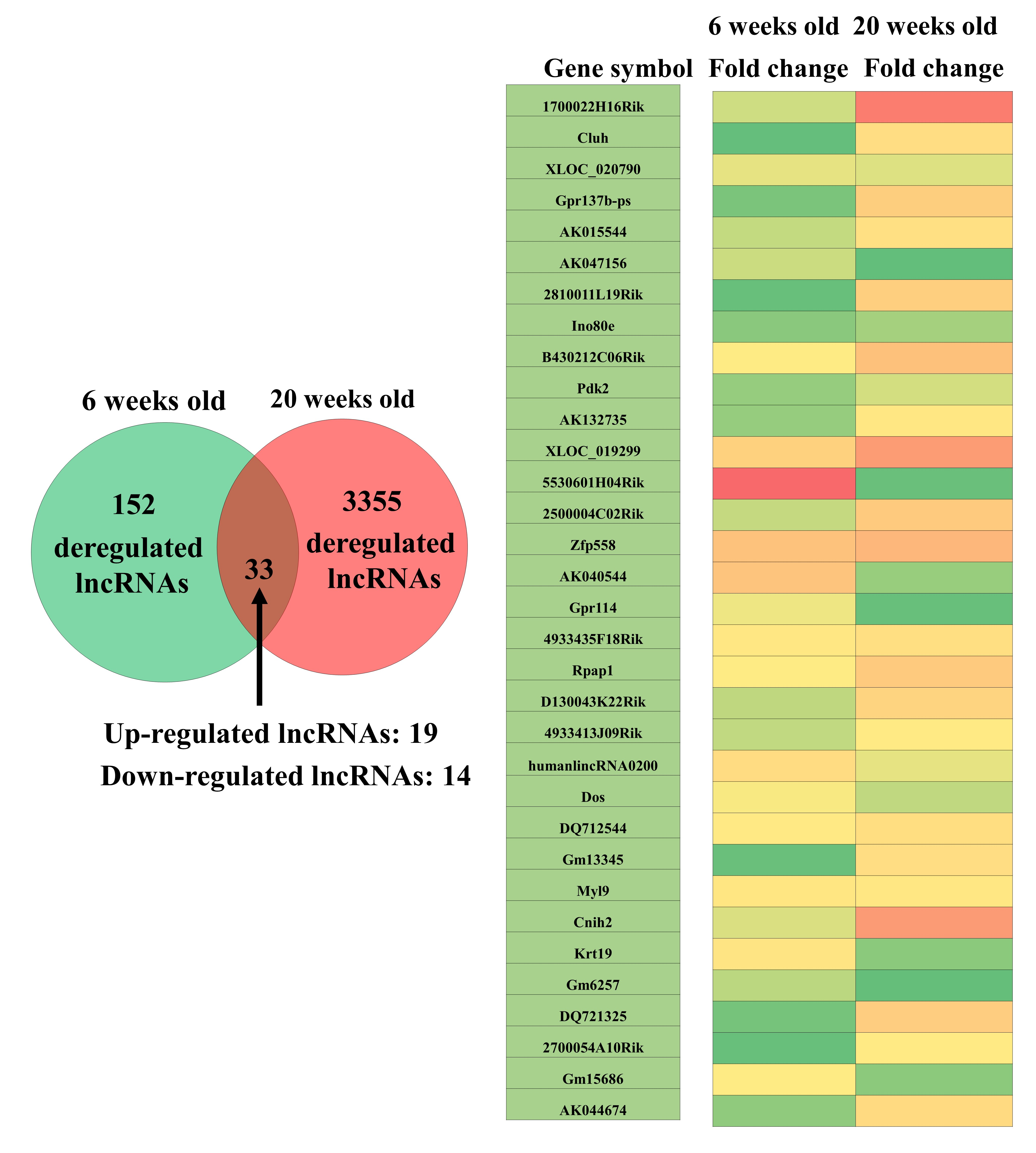


**Figure S1. Overlapping deregulated lncRNAs in db/db mice at 6 and 20 weeks of age.** Colors of red and green represented up- and down-regulated lncRNAs with changes larger than twofold, respectively.

The pathway analysis of the overlapping deregulated lncRNAs between 6- and 20-week-old mice revealed that they were associated with insulin secretion, hypertrophic cardiomyopathy, dilated cardiomyopathy, and cardiac muscle contraction, as depicted below.

#
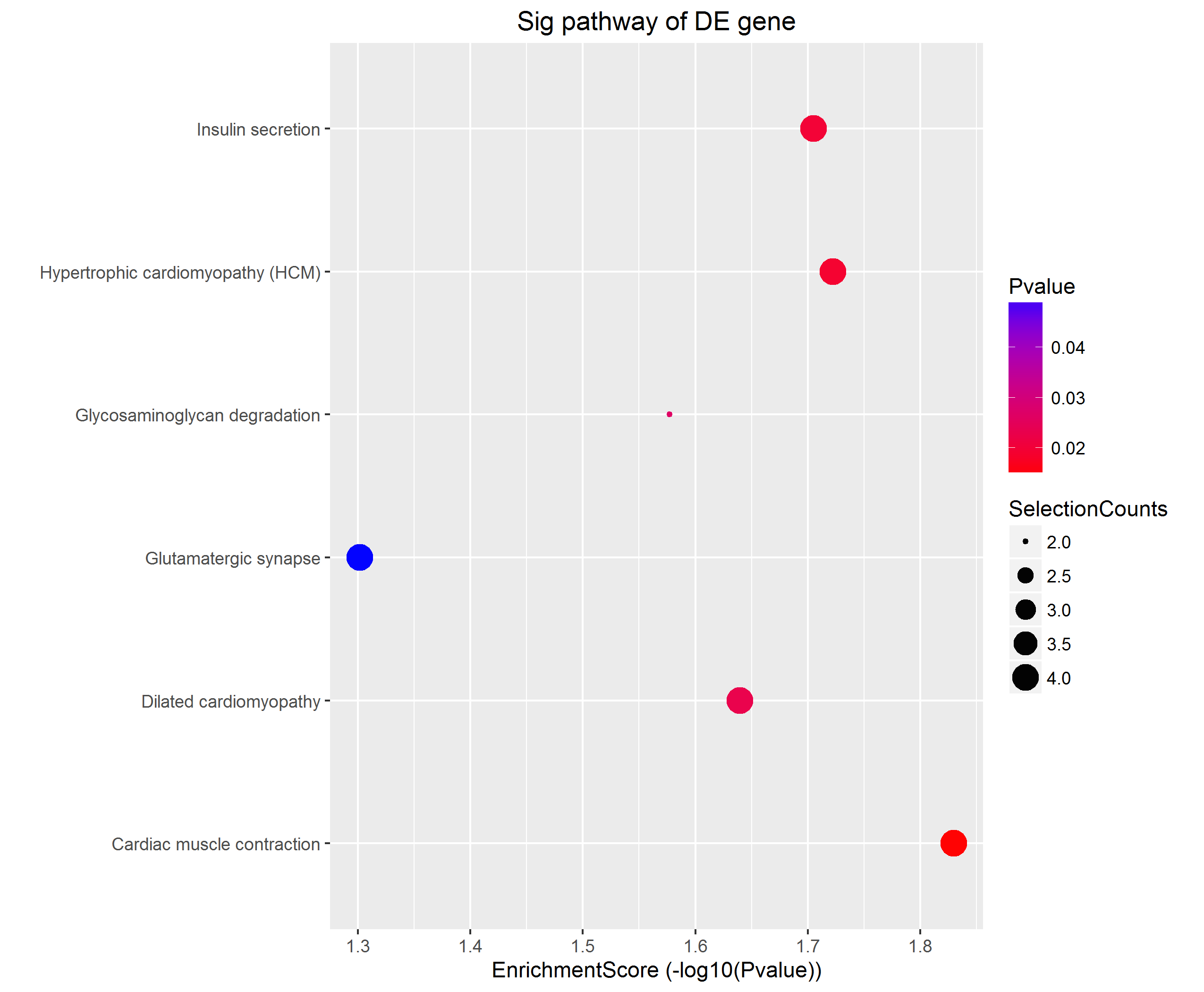


# Annotation of deregulated circulating lncRNAs in db/db mice

The lncRNA can be categorized into intergenic, intronic antisense, natural antisense, bidirectional, exon sense overlapping, and intron sense overlapping groups based on their relative position to the coding gene^9^. Figure S2 shows the percentage of deregulated circulating lncRNA category in db/db mice with and without DCM. In db/db mice at 6 weeks of age, 40% of deregulated lncRNAs were intergenic, 32% were exon sense overlapping, and 11% were intronic antisense (Figure S2A). However, in db/db mice at 20 weeks of age, intergenic, exon sense overlapping, and intronic antisense lncRNAs accounted for 46%, 32%, and 7%, respectively (Figure S2B).


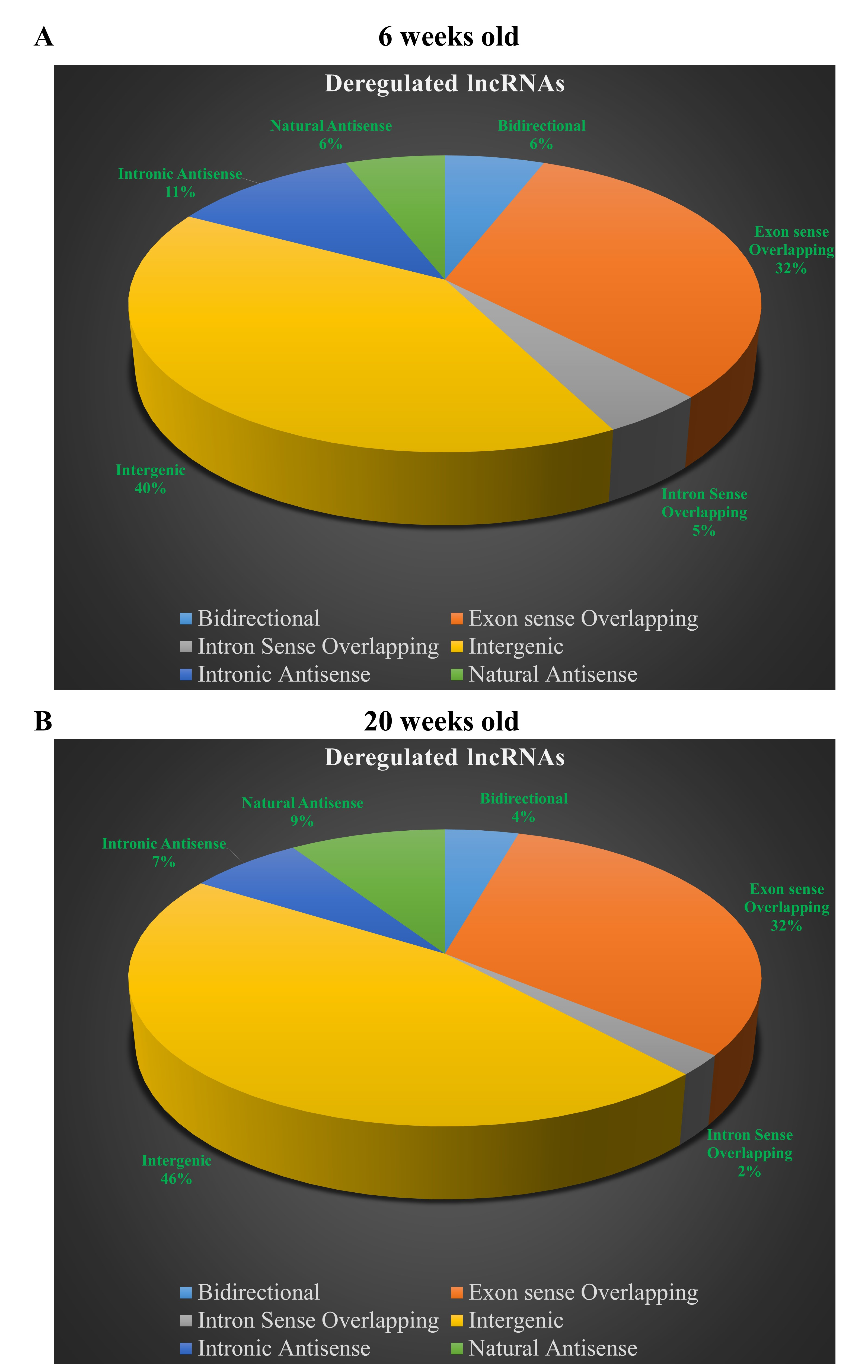


**Figure S2.** **Classification of deregulated circulating lncRNAs in db/db mouse hearts with and without early diabetic cardiomyopathy.** A: percentage of lncRNA categories in 6-week-old db/db mouse hearts; B: percentage of lncRNA categories in 20-week-old db/db mouse hearts.

### **Functional enrichment analysis of deregulated circulating mRNAs in db/db mice**

Figure S3 shows GO enrichment analysis of differentially expressed mRNAs associated with deregulated circulating lncRNAs in db/db mice at both 6- and 20-week-old db/db mice. The up-regulated mRNAs associated with biological processes were biosynthesis and activation of mineralocorticoid and aldosterone in 6-week-old db/db mice and actin-myosin filament sliding and muscle filament sliding in 20-week-old db/db mice, respectively (Figures S3A and S3B). The up-regulated mRNAs associated with cellular components and molecular function were lipoprotein binding and uptake, mitochondrial respiratory chain, and Ca^2+^- and K^+^-channel proteins in 6-week-old db/db mice and myofilaments in 20-week-old db/db mice, respectively (Figures S3A and S3B). Meanwhile, the down-regulated mRNAs associated with biological processes were low glucose import in response to insulin and regulation in both 6- and 20-week-old db/db mice (Figures S3C and S3D). The down-regulated mRNAs associated with cellular components and molecular function were uropod, clathrin-coated endocytic vesicle membrane, and cell trailing edge and store-operated Ca^2+^ channel activity in 6-week-old db/db mice and flotillin complex and insulin-like growth factor receptor binding in 20-week-old db/db mice, respectively. Notably, based on GO terms, altered myofilaments are important in the progression of DCM from diabetes.


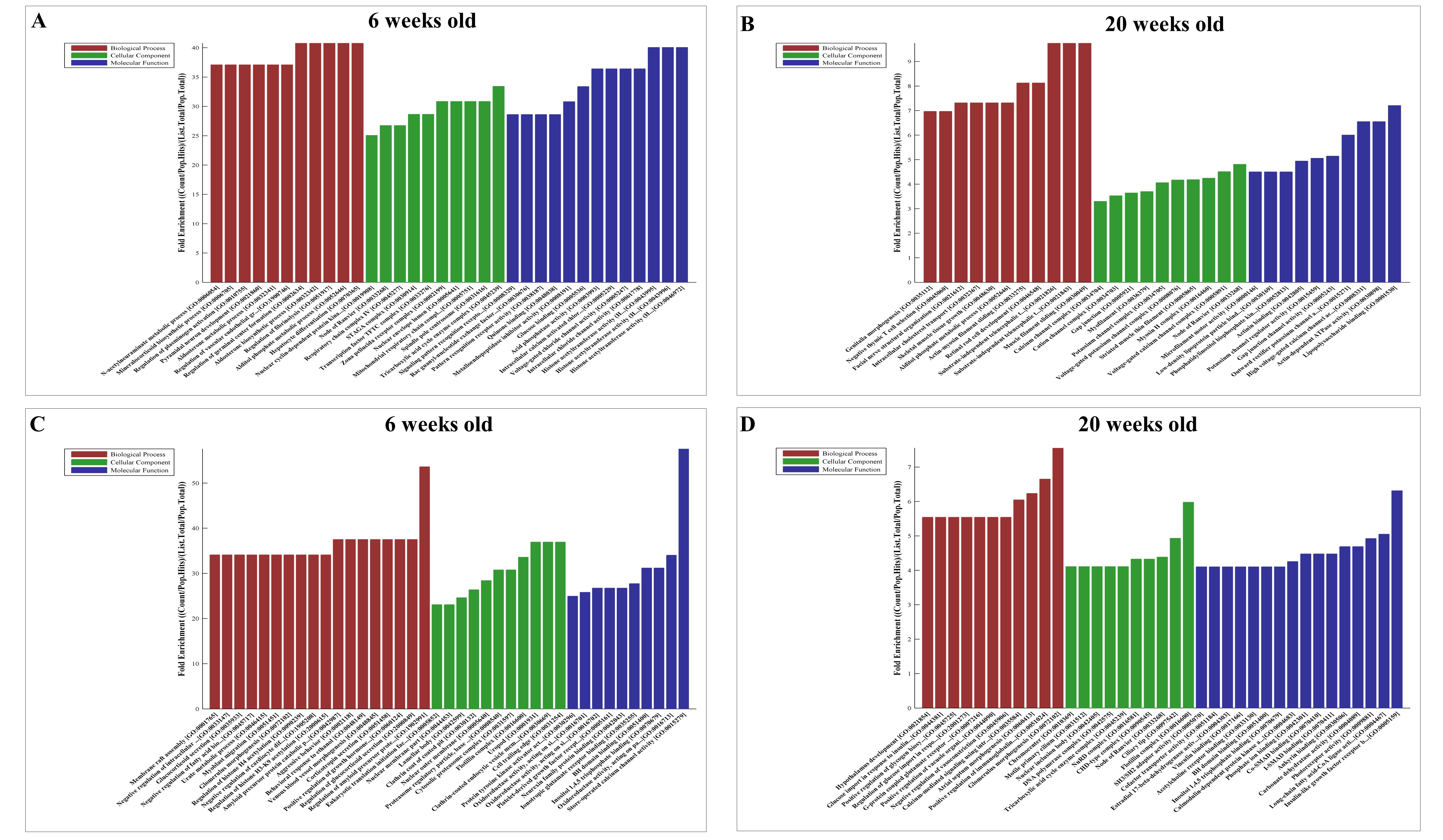


**Figure S3. Gene ontology (GO) enrichment of deregulated circulating mRNAs in db/db mice with and without diabetic cardiomyopathy.** A and B: significantly enriched GO terms of top 30 up-regulated mRNAs in db/db mice at 6 and 20 weeks of age, respectively compared with controls; C and D: significantly enriched GO terms of top 30 down-regulated mRNAs in db/db mice at 6 and 20 weeks of age, respectively compared with controls. Db/db mice had diabetic cardiomyopathy at 20 weeks of age.

KEGG pathway enrichment analysis was performed on differentially expressed mRNAs to reveal the pathways and molecular function related to deregulated, circulating lncRNAs in db/db mice. We found 8 and 10 pathways associated with upregulated mRNAs in 6- and 20-week-old db/db mice, respectively (Figures S4A and S4B); and 5 and 9 related to downregulated mRNAs in 6- and 20-week-old db/db mice, respectively (Figures S4C and S4D). KEGG pathways with significant enrichment of these pathways include tumor necrosis factor (TNF) signaling pathway, mitogen-activated protein kinase (MAPK) signaling pathway in 6-week-old mice and adipocytokine signaling pathway, MAPK signaling pathway, and insulin signaling pathway in 20-week-old mice. As shown in Figure S4B, the adipocytokine signaling pathway and MAPK signaling pathway were the top up-regulated pathways, whereas the top enriched KEGG pathway was insulin signaling pathway for downregulated transcripts in 20-week-old db/db mice (Figure S4D). Based on KEGG analysis, TNF-, MAPK-, and insulin-signaling pathways are critical to the progression of DCM.


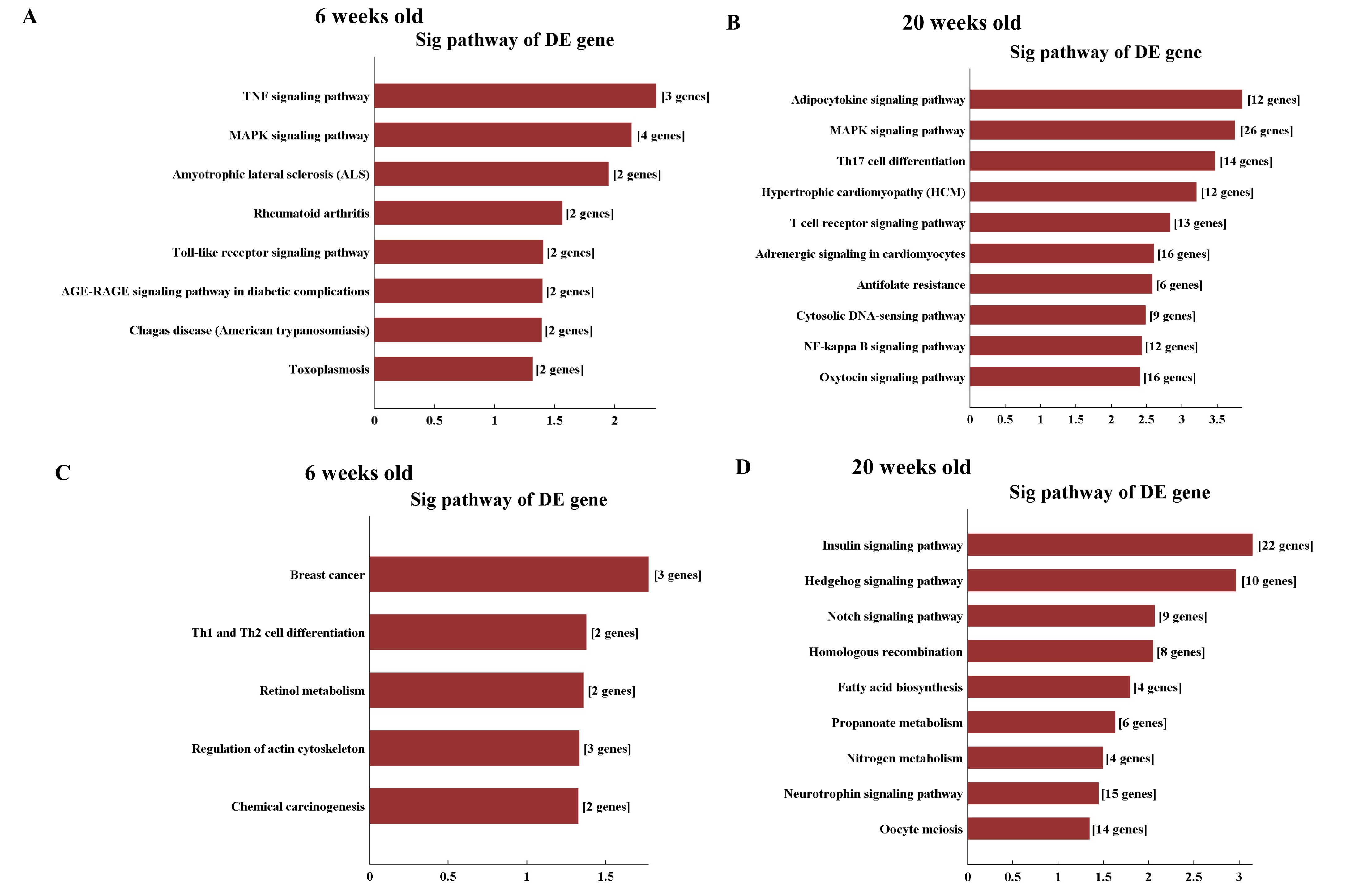


**Figure S4. Kyoto Encyclopedia of Genes and Genomes (KEGG) pathway analysis of deregulated circulating mRNAs in db/db mice with and without diabetic cardiomyopathy.** A: the top 8 KEGG pathways of significantly up-regulated mRNAs between 6-week-old db/db and control mice; B: the top 10 KEGG pathways of significantly up-regulated mRNAs between 20-week-old db/db and control mice; C: the top 5 KEGG pathways of significantly down-regulated mRNAs between 6-week-old db/db and control mice; D: the top 9 KEGG pathways of significantly down-regulated mRNAs between 20-week-old db/db and control mice. DE, deregulated; MAPK: mitogen-activated protein kinase; NF-kB, nuclear factor kappa-light-chain-enhancer of activated B cells; Sig: signaling. The KEGG pathway analysis was performed in accordance with the guidelines [www.kegg.jp/kegg/kegg1.html](http://www.kegg.jp/kegg/kegg1.html).

**qRT-PCR analysis of myocardial lncRNAs in db/db mice with DCM**

To examine how 5 core circulating lncRNAs are regulated in the heart of db/db mice, we used qRT-PCR to analyze their expression in the heart. As shown in Figure S5, the expression levels of the lncRNAs, XLOC015617, AK035192, Gm10435, TCR-α chain, and MouselincRNA0135, were up-regulated in db/db mouse hearts compared with age-matched C57BL/6J ones (P<0.05, n=3/group). These results suggest that myocardial lncRNAs are dysregulated in DCM.

**Figure S5. Expression of myocardial lncRNAs in C57BL/6J and db/db mice at 20 weeks of age.** ^#^P<0.05 vs. C57BL/6J mice (n=3/group).

**Changed myocardial TNF-α and p38 MAPK proteins in db/db mice with DCM**

The bioinformatic analysis revealed the importance of TNF and MAPK-signaling pathways in DCM for deregulated circulating lncRNAs (Figures 4 and S4). To validate this finding, we measured the expression of TNF-α and p38 MAPK proteins in the heart of db/db mice with DCM. Figure S6 shows the representative Western blot bands of TNFα, p38 MAPK, p-p38 MAPK, and GAPDH as control in C57BL/6J and db/db mice at both 6 and 20 weeks of age. Compared with age-matched, non-diabetic C57BL/6J mice, the ratios of TNF-α/GAPDH and p-p38 MAPK/p38 MAPK were significantly increased in db/db mice at 20 weeks of age (P<0.05, n = 5 mice/group) (Figure 7).


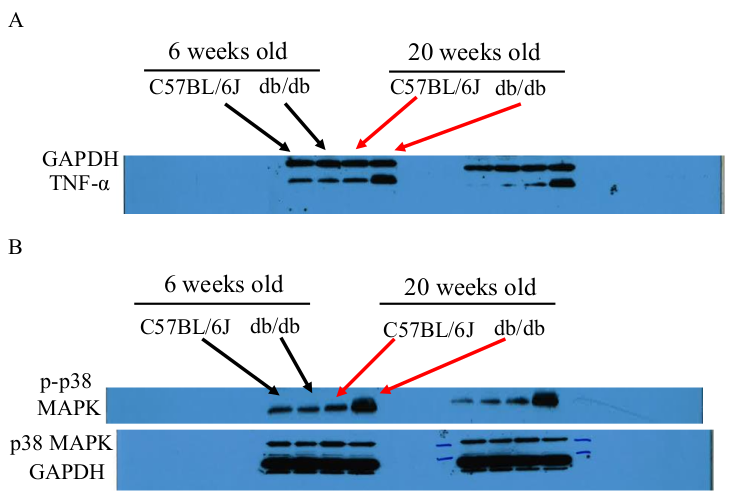


**Figure S6.** **Representative Western blot bands showing the expression of myocardial TNF-α, p-p38-MAPK, p38 MAPK, and GAPDH as control in C57BL/6J and db/db mice at both 6 and 20 weeks of age.**

**References**

1. Pant, T.*, et al.* Genome-wide differential expression profiling of lncRNAs and mRNAs associated with early diabetic cardiomyopathy. *Sci Rep* **9**, 15345 (2019).

2. Wu, H.E.*, et al.* Cardiomyocyte GTP cyclohydrolase 1 protects the heart against diabetic cardiomyopathy. *Sci Rep* **6**, 27925 (2016).

3. Bensley, J.G., De Matteo, R., Harding, R. & Black, M.J. Three-dimensional direct measurement of cardiomyocyte volume, nuclearity, and ploidy in thick histological sections. *Sci Rep* **6**, 23756 (2016).

4. Baumgardt, S.L.*, et al.* Chronic co-Administration of sepiapterin and L-citrulline ameliorates diabetic cardiomyopathy and myocardial ischemia/reperfusion injury in obese type 2 diabetic mice. *Circ Heart Fail* **9**, e002424 (2016).

5. Liu, Y.*, et al.* Transgenic overexpression of GTP cyclohydrolase 1 in cardiomyocytes ameliorates post-infarction cardiac remodeling. *Sci Rep* **7**, 3093 (2017).

6. Cury, D.P.*, et al.* Morphometric, quantitative, and three-dimensional analysis of the heart muscle fibers of old rats: transmission electron microscopy and high-resolution scanning electron microscopy methods. *Microsc Res Tech* **76**, 184-195 (2013).

7. Qiao, S.*, et al.* MicroRNA-21 mediates isoflurane-induced cardioprotection against ischemia-reperfusion injury via Akt/nitric oxide synthase/mitochondrial permeability transition pore pathway. *Anesthesiology* **123**, 786-798 (2015).

8. Liu, Y.*, et al.* Vascular endothelial growth factor regulation of endothelial nitric oxide synthase phosphorylation is involved in isoflurane cardiac preconditioning. *Cardiovasc Res* **115**, 168-178 (2019).

9. Bar, C., Chatterjee, S. & Thum, T. Long noncoding RNAs in cardiovascular pathology, diagnosis, and therapy. *Circulation* **134**, 1484-1499 (2016).
